# Concepts in Alpine Plant Ecology

**DOI:** 10.3390/plants12142666

**Published:** 2023-07-17

**Authors:** Christian Körner

**Affiliations:** Department of Environmental Sciences, University of Basel, Botany, 4056 Basel, Switzerland; ch.koerner@unibas.ch

**Keywords:** climate, biodiversity, ecological theory, high elevation, mountains, niche concept, productivity, reproduction, stress, topography

## Abstract

The alpine life zone is perhaps the only biome that occurs globally where mountains are high enough. At latitudinally varying elevation, the alpine belt hosts small stature plants that vary greatly in morphology, anatomy and physiology. In this contribution, I summarize a number of principles that govern life in what is often considered a cold and hostile environment. The 12 conceptual frameworks depicted include the key role of aerodynamic decoupling from free atmospheric climatic conditions, the problematic concepts of limitation and stress in an evolutionary context, and the role of developmental flexibility and functional diversity. With its topography driven habitat diversity, alpine plant diversity is buffered against environmental change, and the multitude of microclimatic gradients offers ‘experiments by nature’, the power of which awaits multidisciplinary exploration.

## Why Care for Concepts?

As any other field of scientific research, alpine plant ecology is embedded in certain concepts, theoretical frameworks, or paradigms. These emerge from research history, belong to certain research communities, and often simply reflect the fact that some aspects of alpine plant life are understood better than others because methods were and are available. Indeed, the availability of tools often shapes scientific paradigms [1]. It seems useful to revisit some of the existing concepts and ask whether they just reflect legacies or available methods or rest on facts and robust theoretical backing. In this contribution, I depict a number of such concepts, explain their implications, reflect on their acknowledgment in the research community, and discuss future research needs to sharpen their theoretical foundation.

Not a concept, but rather a matter of definition or convention, ‘alpine’ represents the naturally treeless belt above the montane belt, both separated by the high elevation, climatic treeline [2]. Note the difference between this biogeographic definition of alpine and the common-language meaning that often substitutes mountain by alpine (e.g., alpine skiing, alpine cities). There are no cities at alpine elevations (that is, above the treeline) anywhere on the globe, although it often may look like there are, when the montane forest had been destroyed by land use, with trees absent from treeline, a source of confusion. Since the potential position of the treeline can be predicted by climatic data [3], the biogeographic delineation of ‘alpine’ also rests on climatology, and comprises 2.6% of the global land area outside Antarctica [4,5].

Across the treeless alpine world from arctic–alpine to tropical–alpine conditions, plant life is ruled by a number of common drivers, each belonging to different scientific domains and thus rarely considered jointly, given the disciplinary barriers. The sequence of the 12 concepts depicted has no particular meaning, but I start with plant morphology and the physical environment, then move to more physiological aspects, and close with population- and community-related concepts. For the sake of coherence, this overview is restricted to angiosperms, thereby not neglecting the rich alpine cryptogam flora (bryophytes, ferns, lichens) that would deserve a separate assessment. This selection of concepts and their meaning leans on earlier works as summarized in Körner [6,7].

## 1. ‘Small Is Beautiful’—The Life Form Concept

All plant species belong to certain growth forms (the genotype) which become shaped to certain life forms by the environment (the phenotype). While the genotype–phenotype contrast can be large in the case of trees, which could be forced to small shrubs by the environment above treeline (krummholz), the growth form–life form difference becomes increasingly smaller with elevation above treeline. In a first approximation, the life form exhibited by most alpine plants is quite close to their growth form, meaning that transplantation to a warmer, lower elevation location does not fundamentally change appearance, but depending on species, plants may become slightly taller, without losing the architectural principles that control their stature (morphology). Because the life form is what we actually see, I use this term instead of growth form in the following text, with growth form implicitly included (definitions in the tradition of A. von Humboldt, C. Troll and W. Rauh, in [7].

**Figure 1:**
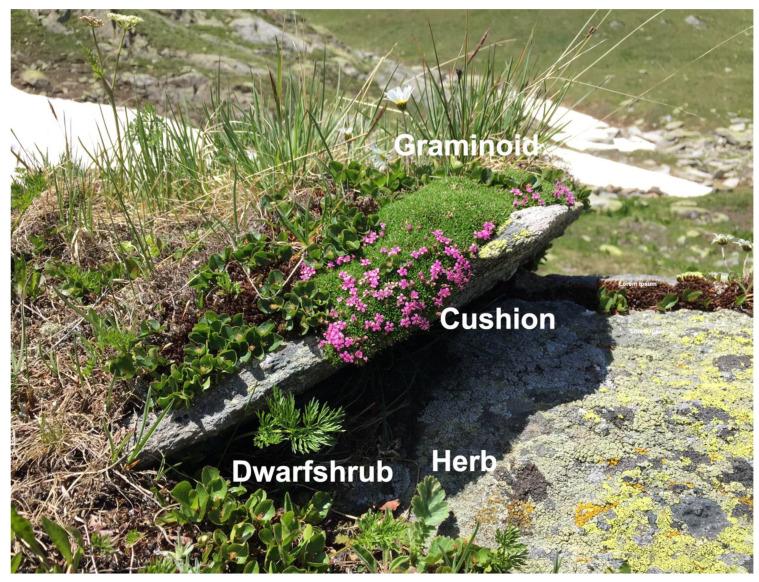
Various alpine plant life forms assembled around a rock in the Swiss central Alps at 2440 m elevation. The graminoid life form is represented by *Nardus stricta* and *Juncus trifidus*, cushions by *Silene acaulis* ssp. *exscapa*, dwarf shrubs by the creeping *Salix herbacea* and herbs by *Geum montanum*, *Leontodon helveticus* and *Ligusticum mutellina*.

As a unifying principle, plants above the climatic treeline are small ‘by design’ (genotypically) and in a large part belong to the four life forms: dwarf shrub, mostly tussock-forming graminoids, mostly rosette-forming herbs and cushion plants [7,8,9] (Figure 1). The restriction of alpine plant life to these low-stature architectures is the outcome of evolutionary selection of taxa that must meet several requirements: (a) decoupling aerodynamically from ambient air conditions as much as possible, and thus engineering a warmer micro-environment close to the ground, (b) restricting apical meristems (buds) to the buffered atmospheric conditions near the soil surface or to below the soil surface (escaping severe freezing), (c) ensuring snow cover in winter (at extratropical latitudes) in species in which winter temperatures above snow would be fatal, (d) guaranteeing mechanical robustness to disturbances by strong below-ground (clonal) structures that are ensuring long life and represent a buffer against the failure of sexual reproduction, (e) enabling rapid seasonal development (leaf dynamics, flowering, seed maturation). At all latitudes, these requirements become increasingly critical with increasing elevation, hence plant species become smaller the higher they are located, and the clonal life strategy becomes increasingly important as sexual reproduction becomes riskier. The life form concept and its implications unify the alpine flora globally. Neglect of the ‘small by design’ principle leads to substantial misconceptions such as the idea that alpine plants are small because they are unable to grow taller phenotypes under the given life conditions [10]. There are numerous other environments that opt for small stature life forms for different reasons, such as pastures, ruderal habitats, steppe, deserts, coastal habitats, salt marshes, fens and mires.

## 2. Habitat Mosaics That Matter—The Role of Topography and Relief

**Figure 2:**
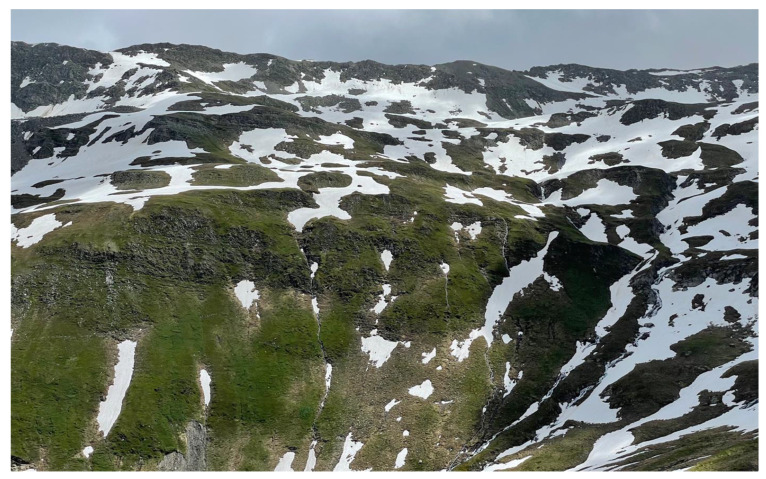
Topography shapes alpine habitat diversity, exemplified here by snow distribution patterns in spring. After snowmelt, this slope exhibited a range of seasonal mean plant temperature of 5 to 14 °C [16]. Swiss central Alps, 2500 m.

In addition to life form, the diversity of microhabitats resulting from exposure to sun and wind, slope inclination, landscape fragmentation by relief (geologically, by periglacial processes and by erosion) creates a multitude of life conditions at very close proximity (often less than a meter). At extratropical latitudes, these land surface properties produce rather diverse snow distribution and thus snowmelt patterns (e.g., [11,12] (Figure 2). At all latitudes, moisture, nutrients and soil organic matter differ substantially across these habitat mosaics, with each of these micro-habitats selecting for assemblages of certain plant species [13,14,15].

A central implication of the concept of habitat diversification at alpine elevations is the unsuitability of elevation-related explanations of current and projections of future plant distributions. Topographic diversity by far outranges elevation-specific changes in environmental conditions [9,15]. For instance, air temperature declines with altitude by c. 0.55 K per 100 m. In contrast, seasonal mean temperatures of alpine plant meristems on a single slope have been shown to vary between 5 and 14 °C at the same elevation (Figure 2) and thus the same mean air temperature in temperate, arctic and high-arctic landscapes [16].

## 3. A Decoupled World—The Overarching Role of Microclimate

**Figure 3:**
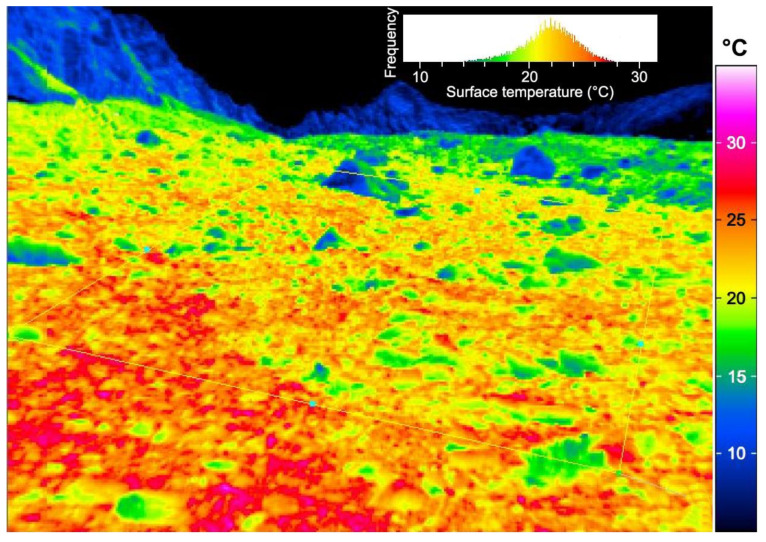
The microclimate in alpine plant communities varies strongly over very short distances, as exemplified by an infra-red thermal image taken in midsummer at 2460 m elevation in the Swiss Alps (Furkapass). Note that the frequency distribution shows a temperature range from 12 to 28 °C, a range that diminishes if averaged over an entire sunny day. However, thermal microhabitat diversity, corresponding to several hundred meters of altitude-related air temperature differences, is retained, and thus disqualifies air temperature data from weather stations for making a case for life conditions of alpine plants (see [16]; photograph by C. Mullis).

As the result of small plant size and often compact morphologies (life forms) on one hand and topographic shelter on the other hand, the actual microclimate alpine plants experience has very little in common with what a weather station or a climate data base would deliver for a given elevation (Figure 3). The substantial physical, that is, aerodynamic decoupling of alpine plants from atmospheric conditions is the central factor in alpine plant life [7,17,18,19]. It comes as a surprise to many when they realize that alpine plants at supposedly cold locations show tropical temperatures in their leaf canopy under bright weather conditions. The interaction of solar radiation with a high aerodynamic boundary layer resistance to heat transfer does indeed cause heat stress problems in some alpine plants. Not surprisingly, the heat resistance of compact alpine plants is quite high (commonly >50 °C, up to 60 °C; [7,20,21,22].

The life-form- and topography-driven microclimate is the gateway to understand alpine plant life. It explains why the photosynthetic temperature optimum of alpine plants (including plants from most extreme summit habitats such as *Ranunculus glacialis* above a 3000 m elevation in the Alps) does not differ from that of their low-elevation relatives [23]. It also explains why >80% of all roots and meristems are located in the top 10 cm of the soil profile, where the heat accumulates. Large inflorescences heat up by 10 K or more above air temperature when the sun is shining [24]. The idea that alpine plants are living in a critically low-temperature world is simply biased by weather station data and hiker experiences [25] and is not based on ground-truth data [7,16]. This divergence of plant and air temperature complicates interpretations of alpine plant performance without in situ temperature records.

The good news is that it was never as simple as it is today to obtain accurate data about the actual life conditions of plants in most remote places [26,27]. Miniature data loggers provide year-round information about the thermal regime experienced. Such signals also indicate presence or absence of snow. It is hard to understand why so much speculation about alpine thermal life conditions becomes published (assuming air temperature to reflect plant temperature) while it is so simple to assess the truth. A functional alpine plant ecology without ground truth data is ill-founded, and the use of climate data obtained from weather services is a no-go in light of what we know (for a summary, see [7,25]. Therefore, conceptualizing alpine plant life in a microclimate framework is imperative. In the absence of on-site data, an empirical variance term of likely plant and soil temperature for a given mean air temperature can be applied to scale from air temperature to a proxy of plant temperature [16]. Spaceborne remote-sensing can also provide proxy data, although such data commonly have limited temporal resolution and are affected by clouds.

A note on facilitation: The microclimate effects explained above rest on aerodynamic boundary layer phenomena related to structural density (leaf area density, LAD; [7] near the ground, irrespective of species diversity. Also, single clonal species such as grass tussocks ([28], mat-forming dwarf shrubs [29], or cushion plants [7] show dramatic microclimate benefits (a sort of ‘self-facilitation’). In fact, all late successional plant communities, including tropical forests, exhibit mutual shelter benefits of closed canopies, because all, except pioneer species, evolved embedded in communities. Thus, it is a truism that most alpine plants need neighbors or sheltered niches in order to thrive—classical wisdom that can also be framed in a ‘facilitation’ concept [30]. Hosting of non-cushion plants in cushion plants is a facilitation that goes beyond traditional micro-climatology [31] in highly eroded and seasonally dry alpine habitats, and this can rise local species diversity.

## 4. From Opportunism to Internal Clock—The Key Role of Developmental Controls

Growth and development are two different categories of life processes, with the latter controlling the former and exerting overarching functions. Development is the genetically controlled transition between plant states such as dormancy, bud break, greening, flowering seed release and senescence. Growth can occur within the ‘windows’ opened by developmental cues transmitted by hormones. The visible part of development is called phenology. These developmental controls are deeply rooted in evolutionary adaptation. Evolution has selected for seasonal dynamics of development that ensure long-term survival under rather unpredictable weather conditions. Whether and how much the day-to-day or year-to-year variation in actual climatic conditions exerts additional influences on plant performance depends on species and life strategies. Species belonging to later successional stages and species of high longevity exhibit stronger genetic control over seasonal activity (phenology) than short-lived pioneer species. Certain environments (e.g., wind edges with insecure snow cover) opt for rather conservative developmental controls, while others (e.g., snow beds) opt for a more opportunistic phenology. The important point is that phenology reflects the long-term mean of season length so that the reproductive cycle can be completed at least in some years, with clonal persistence securing life in other years.

**Figure 4:**
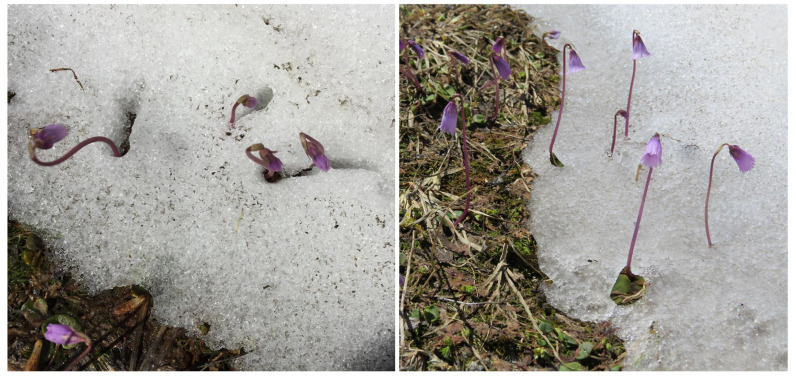
The alpine snow bed plant *Soldanella pusilla* emerges through melting snow in July with fully grown flowering stalks. An internal clock initiates growth in early January under meters of snow pack. Note the spaces around dark plant structures are created by thermal re-radiation (see [11]).

The concept of evolutionarily selected developmental control comes in conflict with ideas that seasonal plant performance is largely driven by concurrent environmental conditions. Therefore, it was predicted that warmer climates will reduce snow duration and thus allow plants to grow for a longer period, with a higher biomass production at community scale. When explored experimentally, this is not what was found in late successional alpine grassland. The most important species of the alpine grassland belt in the Alps, *Carex curvula,* has an internal clock that causes senescence (leaf browning) whenever 6–7 favorable weeks are over, irrespective of the date of release from snow [32]. Therefore, this dominant species makes no use of any extra time. Another form of internal clock is used by the snow-bed species *Soldanella pusilla*. This species grows its complete 5–6 cm flowering stalk and flower from a 1–2 mm bud at snowing-in to the time when it emerges through thin spring snow, ready to welcome bumblebees [11]. Even 3 m under snow, *Soldanella* starts growing in early January (Figure 4). Given the unpredictable day of release from snow, the inflorescence must be ready to perform by late May, even though snow may disappear in early August only, after a snow-rich winter; this is an opportunistic life strategy.

The important point with developmental controls is that short season climates (often at high elevation) opt for rapid development, which requires high rates of metabolism over a rather short period of time, a clear advantage for herbaceous plants, and an obvious disadvantage for woody plants that require far more time to complete seasonal maturation. This is an analogy to the response of hot desert ephemerals to occasional rain events, with the difference that alpine plants can rely on a certain regularity of seasonality and thus have been selected for long life (perennial below-ground structures).

## 5. Persistence Is More Important Than Vigor—A Life Insurance Principle

As mentioned several times above, the clonal life strategy is one of the common denominators among the alpine floras. Just as the life form of a tree, clones of small-stature plants are selected for long life, but in the case of clones, theoretically, for eternal life, year-to-year success of sexual reproduction is not very important. In fact, clonal plants may ‘hold position’ for centuries without sexual offspring. Some genets of late successional alpine clonal taxa have been found to have a life history of several millennia [33,34].

Commonly, such most successful clonal taxa (the majority) grow comparatively slowly on a year-to-year basis, reminding us that evolution does not select for productivity but for fitness, that is, for retaining genes in space over time. In fact, lush (or even uncontrolled) growth is the anti-thesis for persistent life in harsh environments. In other words, the alpine flora makes no exception from the vigor–stress tolerance trade-off that has been described for trees [35]. While seasonal development and short-term above-ground growth may be rater rapid, the controls over its timing (see 4), irrespective of concurrent weather conditions, are part of the survival concept. The exceptionally high investment into below-ground structures may constrain above-ground productivity, but it ensures buffering the effect of adverse conditions [36], including ‘missing a summer’, when snow cover does not disappear in an unfavorable year [11].

Importantly, slow overall growth is not to be confused with low metabolic capacity. It rather reflects a compromise related to longevity, robustness against rapidly changing life conditions, extreme events, dependency in sturdy below-ground (heterotrophic) structures and a need to rapidly complete the seasonal life cycle. With this ‘design’, the majority of alpine plant species is unlikely to track rapid environmental change, with microevolutionary selection of more flexible genotypes likely to take quite some time. I wish to recall that the *Carex curvula* clones examined near our alpine research station at Furka Pass in the Swiss central Alps occupied the same piece of land during the medieval warm period when the Wikings discovered Labrador and people grew grapes in Scotland [33], and during the little ice age, when expanding glaciers endangered mountain villages and trees at treeline hardly grew [37]. The persistence syndrome in late successional alpine plant communities also explains the low sensitivity to ongoing climatic warming in high alpine grassland [38].

## 6. The Many Solutions to The Same Problem—Plant Functional Diversity

**Figure 5:**
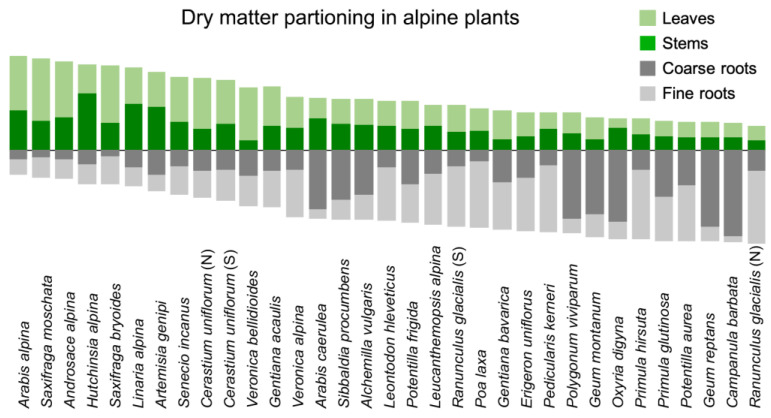
Dry matter allocation in the nival flora of the Alps (data from the central Alps in Tyrol, [40]. The range of allocation patterns covers almost the full known range for angiosperms at sites c. 1000 m above treeline (nival habitats).

There is no ‘archetype’ alpine plant. The morphological, anatomical and physiological diversity is overwhelming even under extremely high elevation conditions [7,39]. In herbaceous plants at >3000 m elevation in the Alps, dry matter allocation (how much biomass is invested in roots, storage organs, shoots and foliage) covers the entire possible spectrum [40,41] (Figure 5), with similar patterns across the globe [7,42]. On the very same square meter, one may find herbs that have 10% or 90% of their total biomass below ground. Similarly, photosynthetic capacity per unit leaf area or the discrimination of the heavy ^13^C isotope (a measure of carboxylation efficiency) cover ranges as wide as can be found in any other environment [23,43,44]. Also, leaf anatomy varies greatly across the globe’s alpine world [45].

In other words, the conditions at high alpine or nival elevation select for traits that are not finding expression in certain common carbon investment patterns, except for reducing plant height, and thus investment in upright stems. Within the life form spectrum present, neither anatomical nor tissue quality traits belong to a common high-elevation syndrome. A delicate small herb may grow next to sturdy sedge tussocks, a dwarf shrub or a semi-woody cushion plant. These patterns clearly falsify (except for small stature) the concept of a common pattern in alpine plant architecture. Different micro-topographies select for higher or lower abundance of different morpho- and physiotypes (e.g., [7,13,46,47,48,49,50], the explanation of which deserves more research. Microtopography-related ‘experiments by nature’ also hold promise for explaining species range limits [51] (see 11). As an example, the graminoid life form is less successful in snowbeds than the life form herb [52], and contrasting snow duration regimes select for certain pheno-rhythmotypes. Persistent clonal structures are abundant across all alpine habitats. It is a truism that alpine plants must be able to cope with the thermal extremes in their habitats (see 8). Therefore, adequate freezing and heat tolerance are a ‘filter’ through which all alpine plants species have to pass, with the consequence that what the successful plant species experience is not stress but part of normal life [9,53]. Yet, their habitat conditions would be rather stressful for non-adapted taxa.

Trait selection may well operate at the earliest life stage (seedling establishment), and not at the life stage at which the commonly measured functional traits of plants are obtained from (see 10). Since it is obvious that alpine plants most commonly require a decoupling from atmospheric conditions (see 3), the type of neighborhood matters as well [54], shifting habitat selection from the individual to the community level. Given the exceptional diversity of the soil microbiome (with thousands of bacterial and fungal organismic taxonomic units, OTUs, obtained from alpine soil samples; M. Grube in [52]), it seems that the microbial diversity covers the requirements of any type of habitat. Even the plants at the coldest known place where plants can live exhibit a rich fungal microbiome [55].

## 7. The Species Diversity–Productivity Relationship Is Confounded

Over the years, the idea became popular that plant species diversity correlates with biomass production. This rationale roots in the valid assumption that species have different resource requirements, and thus a functionally diverse community may extract more from a given resource (e.g., nutrients, light) through the complimentary utilization of a growth-limiting resource (e.g., [56]). Many experiments with designed grassland diversity on common substrates (‘common gardens’) at low elevation supported the concept of the diversity–productivity connection (e.g., [57]). However, when explored in plant communities where species presence was the outcome of natural selection, no such patterns were found [58].

**Figure 6:**
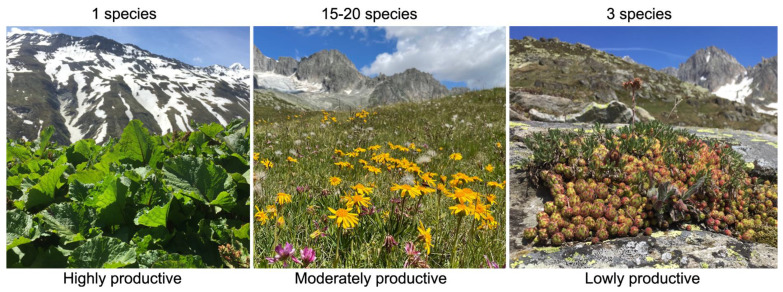
Extremely productive (*Rumex alpinus*) and extremely unproductive (*Sempervivum montanum* with *Leucanthemopsis* alpina and *Leontodon* sp.) communities are species poor, and moderately productive communities are species rich (an alpine grassland with *Carex curvula*, *Nardus stricta*, *Arnica montana* and *Trifolium alpinum*). Swiss central Alps 2440 m (Furkapass).

Under alpine conditions, habitat diversity (and thus very local life conditions) challenges exploring such diversity–productivity relationships. Extremely species-poor communities at harsh locations (wind edges, eroded soils, center of snow beds) exhibit very poor productivity, and highest productivity is found in lush, species-poor or even monospecific stands (e.g., *Carex* fens, *Rumex alpinus* stands on ungulate resting places), with the remaining, closed-cover communities filling the middle ground. When tested within comparable types of grassland, no species diversity–productivity relationship can be found, but a weakly positive trend (r^2^ 0.12–0.19, *p* < 0.01, n = 67) occurs across all non-woody vegetation types in a larger alpine area, provided the above-mentioned extreme cases are excluded [59] (Figure 6). It seems that moderately favorable soil conditions with a moderate productivity facilitate the co-existence of a higher number of species, with a functional interaction between the two rather uncertain. Species diversity–productivity relationships also do not exhibit uniform patterns, and species identity was found to exert a strong influence on productivity in the Tibetian plateau [60]. Thereofre, species diversity and biomass production reflect habitat diversity (soil fertility) while their potential interaction does not match results from common garden experiments where habitat diversity is eliminated and the presence of species is enforced by experimental design.

The functional benefit of high species diversity rather seems to come into play when stochastic external effects or novel threats occur, similar to the role of ‘portfolio diversity’ for buffering shareholder value against financial crises. The persistent plant presence and thus the stabilizing of diverse microhabitats [50] often depend on key-stone species, the functional importance of which becomes obvious under disturbance only. For instance, high species diversity ensures persistent land cover under erosion pressure, as was shown for the Alps and the High Caucasus. A single, common tussock grass species out of many other grassland species became the engineer of erosion edges in both regions. Quite unexpectedly, this extreme situation made this species a keystone species [61,62]. Below-ground diversity was also found key in maintaining grassland integrity after disturbance [63]. Diverse seasonal phenologies are also likely to buffer effects of climatic change (such as changing snow duration) on the integrity and productivity of alpine plant communities [64].

Therefore, alpine species diversity secures continued ecosystem integrity (e.g., preventing erosion) and is rather confounded with productivity than driving it. Habitats with very high species richness fall in the moderately productive category, that is, life conditions that permit co-existence, with no single species overgrowing others. There is a risk that nutrient input (e.g., by ongoing nitrogen deposition) will act as a game changer, favoring a few responsive species at the loss of others (see 12). These few examples illustrate that ‘response traits’ are likely to be ecologically more important than ‘static traits’ (such as leaf size, specific leaf area, tissue nutrient concentration) that might be obtained, for instance, from an herbarium.

## 8. The Concept of Limitation and Stress—Utmost Confusion

It is rather misleading to apply limitation concepts as they were developed in yield-oriented agronomy (Liebig’s law of the minimum) to ecosystem ecology [6,9,53]. Representing the most frequently employed term in ecology, the yield-oriented concept of limitation has no place in ecology. Natural vegetation such as alpine or arctic reflects nature’s answer to the local life conditions. As species assemblages mirror life conditions, any change in these conditions causes the assembly to change. A so-called nutrient-limited alpine grassland turns into a ‘fat’ meadow if fertilizer is added, with all the species believed to have suffered from nutrient limitation becoming locally extinct once relieved from that ‘limitation’.

The concept of plant stress, a severe form of limitation, is a similarly misleading concept [9,53]. A certain degree of stress keeps vigorous neighbors away and ensures persistence for those selected for their stress tolerance. There are lots of anthropocentric interpretations of life conditions that seem hostile for humans. Such conditions are essential for many species that do not tolerate competition but can cope with what by an observer’s rating might be hostile. This does not mean that obviously stress-dominated habitats are providing luxury for those inhabiting it. What might appear like rather supportive physiological life conditions for a species based on its abundance patterns in reality might represent marginal life conditions from a growth physiological perspective, with the abundant species simply coping with these conditions better than others. Often addressed as a discrepancy between the ecological and the physiological ‘optimum’, the first is rated by fitness, the second is rated by biomass accumulation. The latter is unsuitable for making a case for survival and abundance limitations in alpine environments (but possibly also in most other environments). What matters is retaining a species’ gene pool in space over time rather than biomass yield.

Hence, a certain degree of limitation or stress by resources or climate, respectively, is vital for plant existence in the wild. In their natural environment, most plant species operate far away from what might be rated as ‘optimal’ from a growth-physiological point of view. Therefore, the agronomic concept of optimality in terms of productivity needs to be abandoned from plant ecology in general, and alpine plant ecology in particular. The challenge ahead is to identify the degrees of environmental limitations of growth and physiological stress that are essential for keeping a plant’s aggressive neighbors away while at the same time affecting survival and the range limits of species (see 11).

## 9. Alpine Productivity—A Matter of Timing and Ground Cover

Given the low mean ambient temperature and the increasingly shorter alpine growing season at extratropical latitudes, the productivity (biomass yield) per unit land area can be expected to decline with elevation. However, all such comparisons rest on the year as a reference period (the concept of annual net plant biomass production, NPP). What if the actual period of plant growth is accounted for? And what if one accounts for uncovered ground fractions because of rocks or disturbances and refers to productivity per unit of plant covered ground area only?

Such a recalculation of alpine productivity yields a surprise. Per unit of time (per day of growing season) and calculated for full cover areas only, NPP of alpine vegetation does not differ from that of humid low-elevation vegetation (2.2 g d.m. m^−2^ d^−1^ or a mean of 185 g m^−2^ dry matter production over a mean length of the growing season of 84 days across temperate alpine sites; [7,9]. Since the delineation of the actual growing season is very difficult [65], and most of that above-ground biomass production does actually occur during the first 4–6 weeks of the season, the productivity would be twice as high on a ‘per day of actual growth’ basis and thus approaching that of a humid tropical forest. Explanations for this high productivity per unit of time with measurable growth and per unit of fully covered ground include the fact that actual life conditions are not necessarily as bad during the period of active growth as one might expect from elevation (see 3). Physiological adjustments can buffer the remaining thermal limitations, and an efficient below-ground microbiome may compensate for effects of cool soil. Further, in extratropical, high-latitude mountains, nutrients recycled over an entire 12-month period (including continuous heterotrophic respiration under snow [66]) become available during a few weeks of vigorous growth after snowmelt, and stored growth in the form of carbohydrate reserves may also contribute to seasonal above-ground biomass accumulation [36]. The below-ground productivity is likely to be as high as or higher than that above the ground, but it is hard to measure because of the multi-year longevity of all below-ground organs. In the temperate zone, a reasonable estimate of the mean root duration in alpine grassland might be 3–5 years, including roots that recycle within a few weeks of their production and roots that live more than 50 years (e.g., some tap roots). Such estimates of productivity that account for the duration of the growing season and full ground cover falsify the concept of low alpine productivity, provided moisture shortage does not come into play, as is the case in semi-arid subtropical or continental temperate mountains.

**Figure 7:**
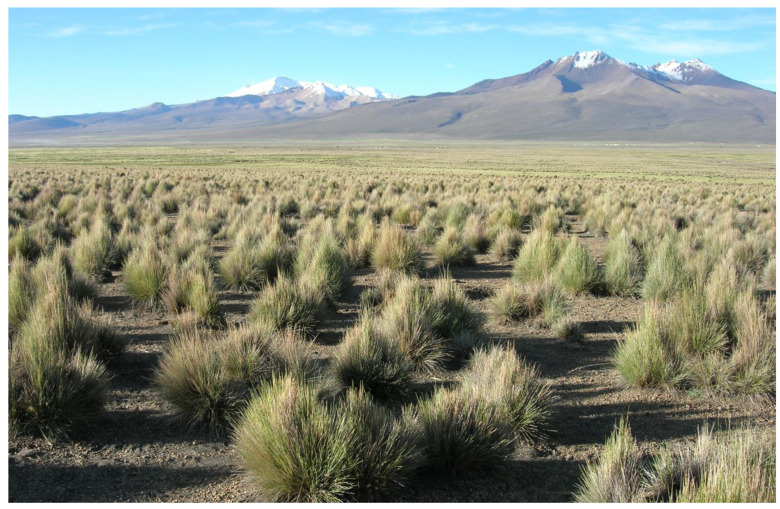
Alpine productivity data must account for ground cover and actual periods of growth. Here is an example of wide spacing of tussock grasses in the Bolivian Altiplano at 4200 m elevation.

Tropical alpine productivity, using a ‘per year concept’ of NPP, is a special and largely unknown case because, similar to root growth in extratropical regions, the continuous growth makes it difficult to identify which biomass was produced in a given year. Seasonally dry tropical alpine settings were thought to represent an exception with a clear ‘rainy season’, but it turned out that wide plant spacing with large root spheres and soil moisture reserves buffer the climatic seasonality, so that growth continues year-round (data from the Bolivian Altiplano by [67] (Figure 7). The only way such above-ground data can be obtained when there is no clear-cut start and end of the season is by assessing shoot (tiller and leaf) turnover by labelling and revisiting. While still neglecting cover effects, these tall altiplano tussocks were estimated to produce c. 1200 g m^−2^ in 12 months [7], which is roughly six times the productivity of grassland in the Alps in a 2-month period of active growth. Repeated harvests are not helpful when growth is continuous. Cutting off biomass may actually stimulate re-growth (as does herbivory) and thus lead to an overestimation of sustainable productivity (compensatory growth; [68,69,70,71]. Therefore, it remains largely unknown whether closed humid tropical alpine vegetation can compensate the cool climate (often very little sunshine, but fog) by year-round growth. Such data are urgently needed to validate the idea that the degree of ground cover and the period of active growth rather than the climatic conditions during this period are the unifying factors that determine alpine productivity.

## 10. The Cradle Is the Bottleneck—Alpine Plant Reproduction

What is it that constrains alpine plant reproduction, that is, the production of reproductively successful offspring (evolutionary fitness)? As explained in Section 5, the overwhelming abundance of clonal life strategies across alpine floras can be interpreted as a safeguard against periodic failure of sexual reproduction. There is also a trade-off between investments in clonal growth and sexual reproduction (e.g., [72]). The high abundance of clonal alpine plants suggests higher risks of sexual reproduction than elsewhere. Yet, the likely risks are manifold, including the loss of flowers by freezing events during the warm season, poor pollination, excessively slow embryogenesis, insufficient seed maturation, seed dispersal, germination and seedling establishment. There is rich literature on each of these steps (summarized in Chapter 16 in [7]). The starting hypothesis is always a concept of serious limitations of each of the various steps till seed dispersal given the assumed hostile life conditions. Surprisingly, all these steps were found to exert no or little risk that would place alpine flowering plants in an inferior position compared to plants from lower elevation, where successful reproduction is not self-evident, either. Even nival plants were found to exhibit no particular pollen limitation [73]. The delicate process of embryogenesis was found to perfectly match even the most extreme nival life conditions in the Alps (e.g., [74] and references to earlier works therein). Whoever explored this was surprised that seeds of most alpine species germinate well, if properly stratified. What is left is the last and much less studied step: the establishment of a new plant from viable seed.

Although not specific to the alpine world, but a crucial step in any environment, the first and second of often very short seasons with a harsh winter, including the risk of low snow cover in early winter and dehydration in summer, provide alpine-specific challenges of seedling survival. It is surprising how little attention this step of the reproductive cycle received compared to pollination and seed viability (but see [75,76,77,78,79]. It may come as a surprise that heat stress is one of the major obstacles that small alpine seedlings have to cope with in bare seed beds (e.g., [22]). Perhaps seedling establishment received less attention because it is a rather tedious fieldwork to track the fate of seedlings under alpine field conditions. However, if we are to understand plant species radiation and species range limits, seed bed ecology needs to be brought to the forefront. Seeds can be found almost anywhere, including very high, exposed summits [27,80,81], but seedling establishment is likely to select for certain response traits and thus species. While summit floras are commonly assembled from species that can grow in isolation or are confined to micro-shelters and tolerate raw substrates, the novel establishment of grassland depends on the success of late-successional species and soil development [82].

## 11. To Be or Not to Be—The Edge of the Fundamental Niche

In my rating, the number one question in plant ecology is why species occur where they do and why they are absent from certain habitats. Once we can explain species distribution, we can attempt mechanism-based projections where species might be occurring when life conditions change. This question boils down to defining the fundamental niche of a species and further to defining and explaining the cold edge of that niche. Given the central role of that question, its neglect in past alpine plant research is surprising (with one first step discussed below). My explanation is the immense task in terms of microhabitat mapping, microclimatology, reproduction biology, stress physiology, life history traits, etc. The edge of the fundamental niche has, for long, not even been assessed for common tree species at low elevation, with a recent attempt ending up in a multidisciplinary mega-project [83], employing a design that also holds promises for alpine plant species range limits. Importantly, the edge of the fundamental niche cannot be assessed by geostatistics or mapping of occurrences, but requires a mechanistic approach, including identifying and explaining ‘extreme’ life conditions.

**Figure 8:**
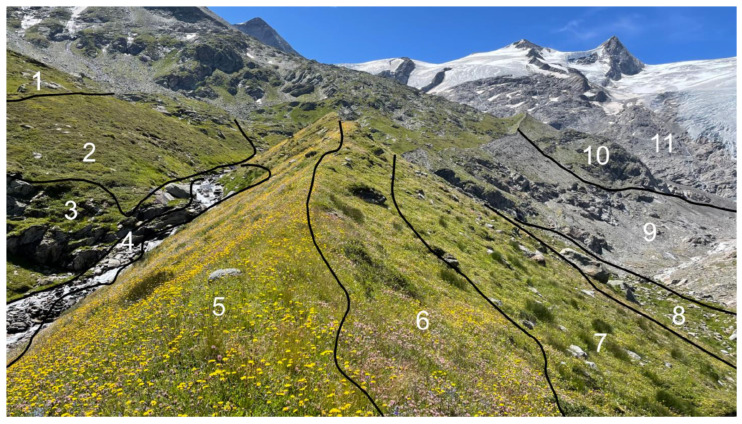
At the same elevation (here, in the Austrian Hohe Tauern National Park at c. 2250 m elevation), topography and associated microclimate and soil conditions shape plant community distribution, challenging the niche concept of species ranges. What we see and what can be mapped is the edge of communities and associated realized niche edges of species (1–11). What is needed for predicting species occurrences for specific abiotic environmental conditions is the edge of their fundamental niche, because it is species rather than communities that shift (though modulated by biotic interactions, to varying degrees).

For explaining alpine plant species distribution, it is essential to know the species’ niche preferences, ideally though difficult, those of the fundamental niche [84,85,86]. Identifying potential range limits of species and the traits that determine that potential edge of their life will open a novel arena of functional ecology. Note that similar to the treeline concept [2,7], this concept is built upon potential performance, with the realized niche (the actual distribution) reflecting distribution history, stochastic events, biotic interactions, disturbances, lack of soil, plant–animal interactions, etc. Since these influences vary from place to place, it seems near impossible to formulate hypotheses and predictions. However, in contrast to the edge of the fundamental niche (representing ‘extreme’ life conditions for a species), the edge of the realized niche can be seen in the field and mapped (Figure 8). We do not know in which alpine species the realized edge matches the fundamental edge, nor do we know for any alpine species how far from its potential range limit it is currently operating. The rapid arrival of new species in previously almost empty summit floras (see 12) points at a substantial leeway for spreading under climate warming conditions, and it also shows which species are more likely to track the spatial shift of their fundamental niche edge.

To the best of my knowledge, the only case in which the range limits of alpine species was nailed down to a mechanistic explanation is that by Von Büren et al. [51]. I think this study adopted a most promising conceptual framework that includes detailed spatial mapping of occurrences, very detailed micro-climatology and a professional stress-physiological assessment, all three possibly depending on a field station very close to the study area. This work revealed that the spatial segregation of the two dominant alpine graminoids in the central Alps, *Carex curvula* and *Nardus stricta*, is controlled by snow distribution in winter, with periodic low or absent snow cover (thus, micro-topography) and the maximum freezing tolerance of the tissues surrounding the apical meristems co-explaining their distribution.

Given the fragmentation of the edge of the fundamental niche to micro-habitats [15], any projections of future species distributions need to rest on a rather fine-grained representation of the alpine landscape. Randin et al., [85] showed that even a 25 × 25 m grid is too coarse to reliably represent the (statistical) spatial distribution of alpine species across two separate test regions. The predicted ‘space-for-time’ loss of species ranged from 20% to 96% for a 3 K warming scenario depending on whether one adopts a 1 m or a 100 m grid [87]. Thus, fine-grain representation of the alpine landscape (thereby accounting for potential micro-topography effects) is essential in modelling alpine plant distribution [88]. The overarching role of micro-habitats as micro-refugia during adverse periods had been illustrated for rock specialists by [89].

## 12. Environmental Change Is ‘Normal’ in Alpine Life

The previous points illustrated that variability in space and time is a unifying factor of alpine environments. This is the matrix in which long-term changes in atmospheric conditions and other anthropogenic influences need to be rated. The term ‘global’ means that the associated changes are considered to apply over large regions, hence they are not region specific. Among these changes, those in atmospheric chemistry (concentrations of CO_2_, reactive N-compounds, ozone) and climate (temperature, humidity, precipitation, snow cover) act globally. Land-use changes, either by intensification or abandonment (e.g., pastoralism, mining, hydrology, tourism), do occur globally, but the intensity of these changes varies greatly among different regions. For earlier assessments of such impacts on the alpine flora, see ([7], Chapter 17; [90,91]). Here, I depict a few aspects of atmospheric changes which are nested in traditional concepts: the concept of carbon limitation and of nitrogen ‘limitation’ (see 8), with a few words on the role of climate warming (for recent statistics of the plant literature on alpine global change, see [92].

As atmospheric CO_2_ rises, plants living in ‘thin’ air (low partial pressure) might be expected to draw particular benefits from that rise, because leaf photosynthesis in alpine plants increases when supplied with extra CO_2_ [23,93], and thus plants might be expected to grow faster (the concept of carbon limitation). By all what we know today, the idea of carbon limitation has been falsified empirically, in situ, for both late successional and pioneer alpine vegetation [94,95]. A complementary test of the C limitation hypothesis is reducing light. When seasonal light consumption was experimentally reduced in half, this also did not affect peak season biomass ([7], Figures 11 and 14; [96]), thus validating the results of CO_2_-enrichment experiments. Not unexpectedly, a 90% reduction in light does, however, exert dramatic effects [97]. However, an important caveat to these bulk biomass responses is that in each case, species-specific responses were observed. In the Alps, one grass species that currently contributes little to bulk biomass profited from CO_2_ enrichment and suffered from shading (*Helictrotrichon versicolor*), pointing at the possibility of long-term community adjustments. To date, these results suggest that alpine vegetation is carbon saturated, but long-term biodiversity effects cannot be ruled out. Once more, responsiveness (response traits) plays a key role. The conventional static traits do not show anything special for *Helictotrichon versicolor* compared to, for instance, *Poa alpina*. This similarly looking pair of species might offer an explanation for what causes a species’ growth to become CO_2_-responsive under alpine life conditions.

Given that photosynthetic capacity does not limit growth (see above) and that temperature hardly affects seasonal CO_2_ assimilation in alpine plants (see 3, [7]), any mitigation of low temperature limitations in a warmer world must act via direct influences on growth and development (including night time effects) and indirectly via controlling season length in extratropical regions. For low-temperature-related growth limitation to come into play, the meristematic tissues must be critically cold. For apical shoot meristems and leaf primordia, this is mostly not the case (see 3), but for deep roots and roots on permafrost, such constraints are possible. It was shown that roots grow very slowly below 5 °C and not at all at 0 °C. The strongest stimulation of alpine root growth by rising temperature occurs between 5 and 10 °C, with little additional benefits at temperatures above 10 °C [98,99]. Overall, soil heating under solar radiation diminishes such limitations, with the remaining influences of temperature indirect, via season length and snow duration.

A wide-spread assumption is that a longer season accelerates development and productivity. Both these assumptions have not been supported empirically in late successional alpine vegetation so far [32,38] (see 8), but a thermophilization of summit floras is evident (e.g., [100,101]. Developmental responses to climatic warming that translate into changes in phenology are rather mixed so far, with some species flowering earlier, and others responding little (see discussion in [7,64]. The concept of a simple tracking of temperature by development has been falsified, and photoperiod controls or internal clocks are important constraints. For climate warming to change development (phenology), micro-evolutionary selection from the existing gene pool would be required, which is another promising field of future research.

Finally, I wish to draw attention to an underestimated global change driver: soluble N-deposition. While all alpine ecosystem (except for cattle resting places) would become more productive (while losing species at the same time; see 8), the ongoing rates of reactive N-deposition are a multiple of pre-industrial background deposition (in essence, lightning effects) and reach 7 kg N ha^−1^a^−1^ in the Swiss Alps [102], with very high rates also reported for the Rocky Mountains and parts of China (for references, see [7], Chapter 17). The effects on plant species are clearly different, with vigorous species, and some sedges in particular, taking more advantage than slow-growing species (e.g., small herbs), leading to a trivialization of the flora ([7] Chapter 17; [103]). Most field experiments with reactive nitrogen addition apply excessively high rates, which might reveal fast responses and a ranking of species responsiveness but are unsuitable to infer long-term effects of nitrogen deposition. This aspect of global change also underpins the misleading nature of the concept of nutrient limitation (see 8).

## Concluding Comments

Extending the famous statement by Dobzhansky [104], ‘Nothing in biology makes sense except in the light of evolution’, one could add for alpine plants: ‘Nothing makes sense in alpine plant biology unless one accounts for micro-climate’. The 12 conceptual frameworks addressed in this paper are certainly not covering all aspects of alpine plant biology, but each of them deserves re-thinking some of the mind models that have been driving much of the research we have seen published over the years.

In my view, research on species range limits (the edge of their fundamental niche) and on genetic variants of existing response traits within species (illuminating where microevolution can select from, under climatic change) deserve high priority. From all what we know about life cycles in alpine plants, seedling establishment appears to be the most critical step in plant species distribution. In light of the many structural and metabolic solutions to cope with the same environmental conditions, static plant traits hold little promise as predictive tools. What is needed are response traits, that is, the way plant species react to local changes in their environment, either naturally or by manipulation experiments.

Among these response traits, the consequences of varying snow cover and the consequences of long-term nitrogen deposition, the differential species responses in particular, deserve far more attention, with existing response indicators for the flora of the Alps awaiting more applications. Expert-knowledge-based ‘indicator values’ proved to be valuable proxies for plant responses to environmental conditions [105,106,107,108,109,110,111,112]. At the community level, such response proxies are often as strong or stronger as on-site physico-chemical assessments of growth conditions.

Given the importance of existing genotypic variation of plant responses to environmental change as well as phenotypic plasticity within genets, experiments that permit assessing both are required. Common gardens for experimentally exploring these fields are one established method, but they bear a risk of bias, because any given common garden provides (a) asymmetric climatic life conditions for species originating from contrasting ‘home’ climates and (b) the common soil represents an asymmetric treatment as well, given the known soil preferences of plant species. Hence, what is considered ‘common’ is in fact an unbalanced treatment. A species from a warm and a species from a cold origin brought to an intermediate location will experience contrasting directions of shift. There is no perfect solution, but replicated common gardens at contrasting conditions can improve the situation, with the central obstacle being the substrate influence. Neither a common (inevitably artificial) substrate nor substrate translocation solve the problem. Substrate fertility may affect all climate response traits. For genotype tests, a most robust approach might be to sample replicated clonal fragments of different genets of the same species and replant them across the species’ home range [113,114,115]. Reciprocal transplantations of entire sods or monoliths can overcome the soil bias, but target species remain tied to the given neighborhood in the plant community. Alexander et al. [54] showed that as species move upslope, effects of species–species interactions can exceed the direct effect of climatic warming on species. Acknowledging and critically applying these concepts in the study of alpine plant life will require more tedious, more complex field work, avoiding over-simplified, seemingly ‘standardized’ experiments in greenhouses, and will bring us closer toward answering ‘big’ questions such as why species grow where they do.

## Data Availability

Not applicable.
